# Single Model for Organic and Inorganic Chemical Named Entity Recognition in ChemDataExtractor

**DOI:** 10.1021/acs.jcim.1c01199

**Published:** 2022-02-24

**Authors:** Taketomo Isazawa, Jacqueline M. Cole

**Affiliations:** † Cavendish Laboratory, Department of Physics, University of Cambridge, J. J. Thomson Avenue, Cambridge CB3 0HE, U.K.; ‡ ISIS Neutron and Muon Source, STFC Rutherford Appleton Laboratory, Harwell Science and Innovation Campus, Didcot, Oxfordshire OX11 0QX, U.K.; ¶ Department of Chemical Engineering and Biotechnology, University of Cambridge, West Cambridge Site, Philippa Fawcett Drive, Cambridge CB3 0FS, U.K.

## Abstract

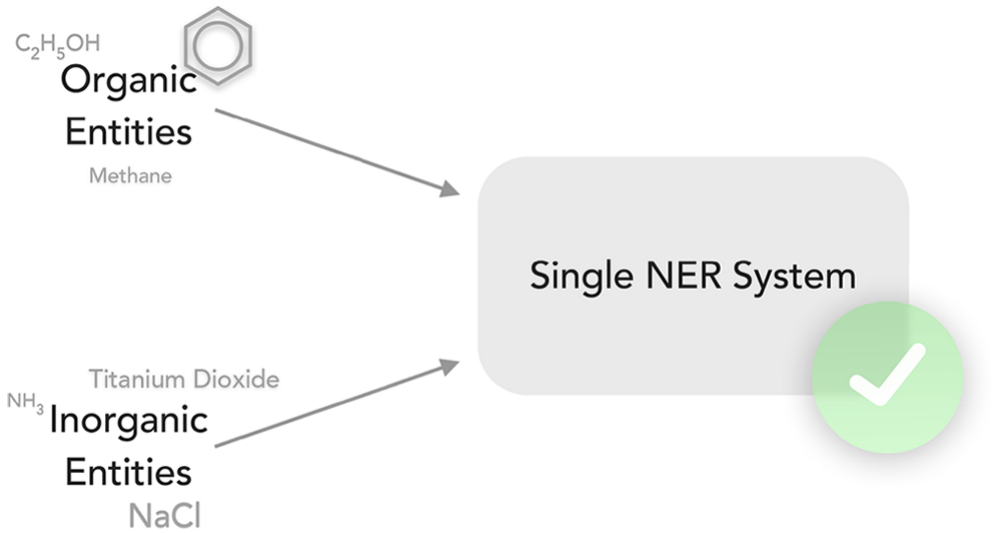

Chemical Named Entity Recognition (NER) forms the basis of information extraction tasks in the chemical domain. However, while such tasks can involve multiple domains of chemistry at the same time, currently available named entity recognizers are specialized in one part of chemistry, resulting in such workflows failing for a biased subset of mentions. This paper presents a single model that performs at close to the state-of-the-art for *both* organic (CHEMDNER, 89.7 F1 score) and inorganic (Matscholar, 88.0 F1 score) NER tasks at the same time. Our NER system utilizing the Bert architecture is available as part of ChemDataExtractor 2.1, along with the data sets and scripts used to train the model.

## Introduction

While vast numbers of papers are written about new discoveries in the chemical domain, the field cannot fully leverage these discoveries, as there are far more papers being published than one could conceivably read. A possible remedy to this situation is the use of automated Information Extraction (IE) systems, such as our previously published versions of ChemDataExtractor.^1−3^ These tools allow for the creation of large databases that can then be used to predict novel material properties.

A fundamental building block of such chemistry-based IE systems lies in the chemical Named Entity Recognition (NER) task. As a large proportion of the properties that one would be interested in involve one or more chemicals, it would be impossible to perform reliable IE without robust NER. There have been a number of data sets that promote the creation of such systems, including the CHEMDNER corpus^4^ for organic chemicals and the Matscholar corpus^5^ for inorganic chemicals.

While there have been systems trained on each of these corpora that perform extremely well, there does not appear to be a system that has been created for cross-domain performance that would work well for the NER of both organic and inorganic chemical entities. For fields involving both types of chemicals, a single-domain oriented NER system would fail to extract a biased subset of chemicals depending on the corpus upon which it was trained, which may result in the creation of a biased data set and therefore biased predictions.

Moreover, even if one was only concerned with extracting information about those fields where the NER theoretically needs to perform well only on one domain, the difficulty of choosing papers that exclusively reference one type of named entity means that the system could extract false positives for papers out of its domain.

To further the state-of-the-art for chemical NER, this paper presents a high performance NER system using SciBert,^6^ a model based on the Bert(7) architecture. The Bert architecture achieves state-of-the-art performance in many tasks, including NER, as shown by its performance in the CoNLL 2003 task.^7,8^ While Bert was trained on a combined corpus formed from the BookCorpus^9^ and Wikipedia, SciBert was trained on the Semantic Scholar corpus.^10^ As a result, SciBert has been shown to achieve improved performance over Bert on scientific tasks.

Following this architecture, the system proposed in this paper achieves close to state-of-the-art performance on organic and inorganic corpora at the same time by training on a combined corpus. We produce a single system that simultaneously achieves high scores on organic and inorganic corpora. Our system attains an F1 score of 89.7, 2.9 percentage points below the state-of-the-art^11^ on the organic-focused CHEMDNER data set,^4^ and an F1 score of 88.0, 2.3 percentage points below the state-of-the-art on the chemical compound recognition subtask of the inorganic-focused Matscholar data set.^5^ While our system does not attain state-of-the-art performance in either subdomain of chemistry, the key result is its high performance across *both* organic and inorganic domains, as this affords it a generalizability that makes it a powerful component for real world IE.

This NER system, along with the data that was used to train it, is openly available, as detailed under Data and Software Availabililty.

## Methodology

**Figure 1:**
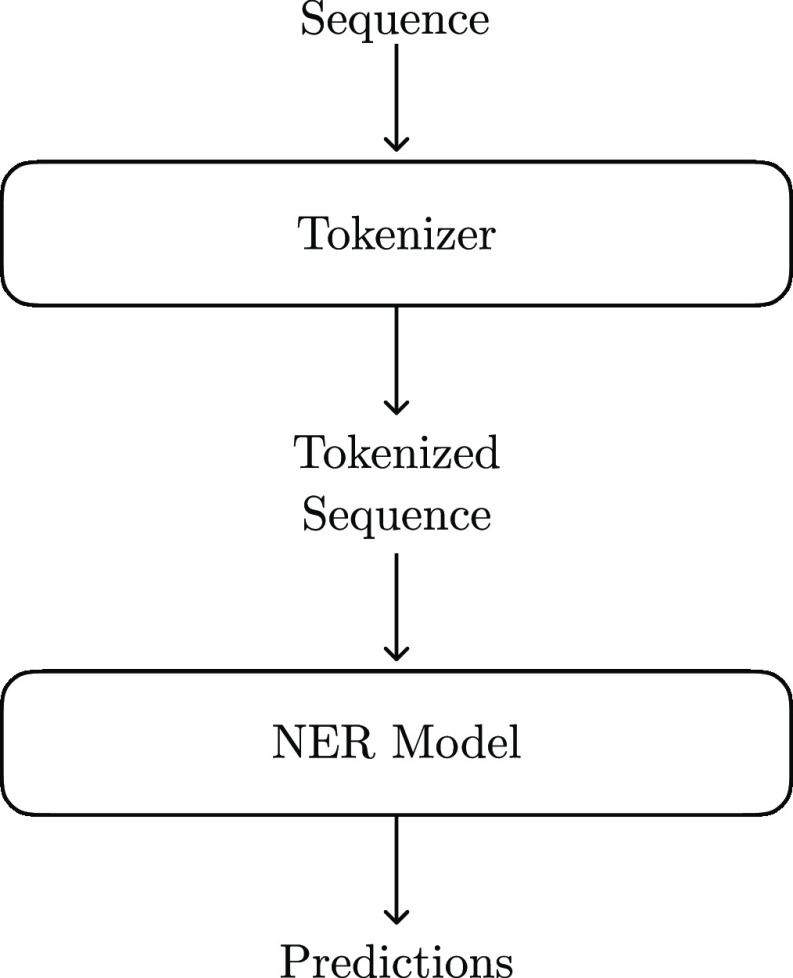
Overarching structure of the proposed NER system. The text is first split into individual tokens by the tokenizer and then processed by a separate model that takes as input the tokenized sequence.

The full NER pipeline proposed in this paper can be seen in Figure 1. In this pipeline, the NER system is composed of two parts: the tokenizer and the model. The tokenizer splits each sentence into individual words, and this tokenized sentence is then fed into the NER model. This section details our methods for each of these two steps.

### Tokenization

As demonstrated by prior research,^12^ the tokenization algorithm can have a large effect on NER performance. We therefore compared two tokenizers that are known to perform well on chemical texts. Analogous comparisons on additional tokenizers are available in the Supporting Information.

#### ChemDataExtractor 1.0 Tokenizer

A natural choice was the tokenizer developed for ChemDataExtractor 1.0. This was based on rules that broadly matched the Penn Treebank policy.^1,13^ This resulted in a high performance for ChemDataExtractor 1.0 on the CHEMDNER data set.^1^

#### Bert Tokenizer

The Bert tokenizer was another natural contender, as our NER model would be based on the Bert architecture. The Bert tokenizer is a WordPiece^14^ tokenizer, meaning that it is a learned tokenizer which is based on the most frequently used word or subword units from the corpus. SciBert enhances this for scientific literature by training on the full text for millions of scientific papers.^6^

As the NER predictions from our model would be at the word level, any subwords from the SciBert tokenizer were merged, and the resulting tokenization was compared with those from the ChemDataExtractor 1.0 tokenizer.

#### Comparing Tokenizers

An objective metric was needed to compare these two tokenizers. To find a suitable metric, what is meant by a good tokenizer must be defined. We define a good tokenizer as being one that tokenizes the text such that tokens do not contain more than two words, while at the same time avoiding the oversplitting of words.

The desirability of these properties can be understood by thinking about a bad tokenizer with the opposite properties. A bad tokenizer that incorrectly outputs tokens containing multiple words would limit the potential NER performance since the model can only predict named entity boundaries that align with the boundaries of the tokens. If a chemical named entity were to be included in the same token as a neighboring word, the correct boundaries for the named entity would not be found no matter how accurate the model may be. In contrast, overtokenization could result in the model having to recognize longer range correlations than if the sentence were appropriately tokenized, reducing performance.

To measure how much these two tokenizers aligned with these two characteristics, we focused on two metrics when applying the tokenizers to the CHEMDNER training set.^4^

The first metric was the number of partial chemical entities. A partial chemical entity, that is to say, an insufficiently tokenized chemical entity, is one where a part of a token was labeled as a chemical entity, but the rest of the token was not. This gives a measure of whether or not tokens contain more than one word. The second metric was the maximum length of a tokenized sentence, a measure of overtokenization. A maximally bad tokenizer would tokenize the text such that each token would contain just one character.

### NER Models

While the cased version of SciBert was used as a fundamental part for our NER model, three different variations of this model were created which used this in different ways, as detailed below. All models were implemented using the AllenNLP framework.^15^

**Figure 2:**
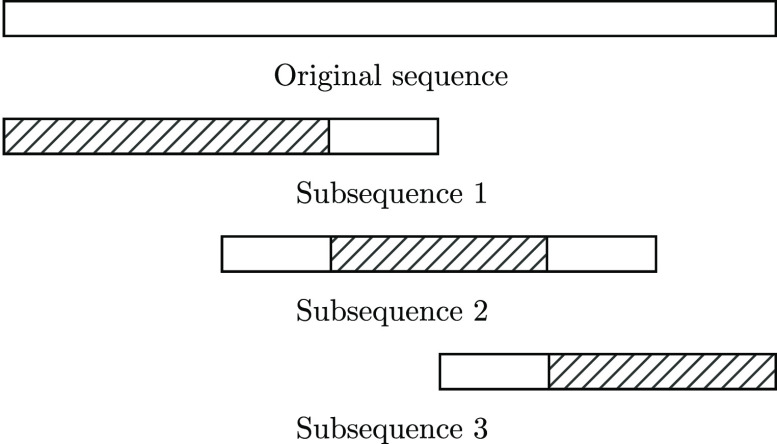
Sliding window approach used to classify sequences longer than those that were used to pretrain SciBert. The original sequence is split into a number of overlapping sequences. The predictions from near the edges of the subsequences are discarded, and the shaded parts of the subsequences represent the parts from which token label predictions are taken for each subsequence.

The SciBert model was only pretrained on sequences that were up to 512 tokens long due to the quadratic nature of attention.^6,7,16,17^ However, the CHEMDNER data set contains sequences that are longer than this limit, so a sliding window approach was taken for all variants to accommodate such sequences. This approach splits an overly long sequence into a number of smaller subsequences, each of length up to *n*, where *n* is a number divisible by 4. To maintain as much context as possible, labels predicted for the first 3/4 of the token are kept for the first subsequence, the labels for the middle 1/2 are kept for intermediate subsequences, and the first *n*/4 labels are discarded from the final subsequence. These predictions are then merged to form the final predictions. This procedure is demonstrated in Figure 2.

#### Fine-Tuning SciBert

**Figure 3:**
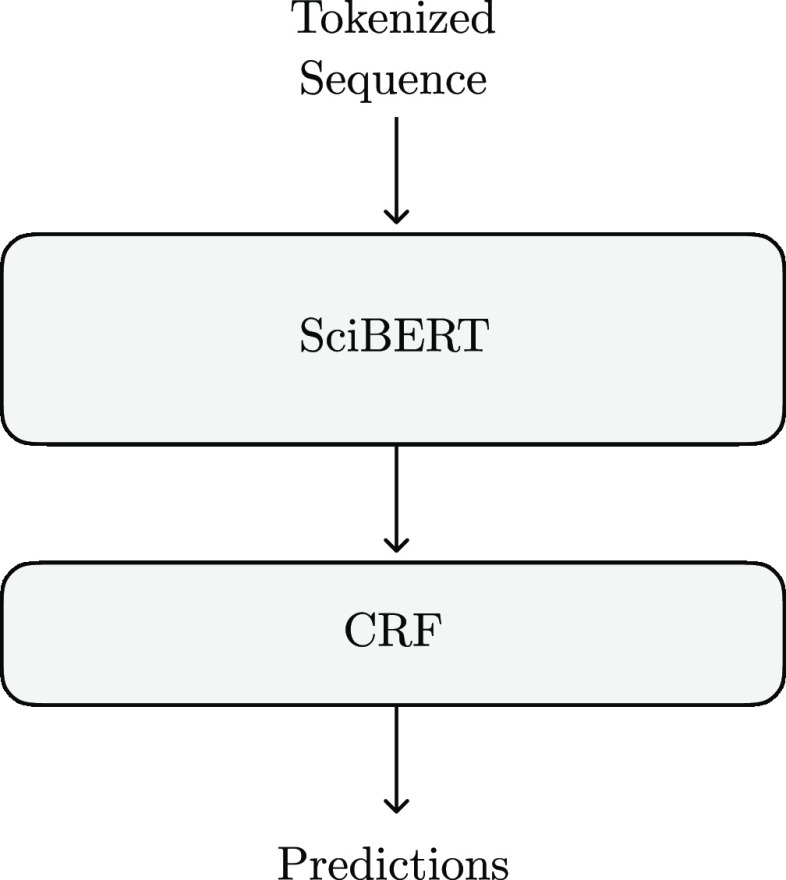
Structure of the NER model created by fine-tuning SciBert.

One approach to performing NER using the Bert architecture is to fine-tune the entire SciBert model to find the named entities. The output from this is fed into a Conditional Random Field (CRF) to ensure valid tags. This architecture can be seen in Figure 3.

#### Using SciBert for Contextual Embeddings

The other approach to performing NER with a pretrained Bert model is to use it as a source of pretrained contextualized word embeddings. To do this, SciBert vectors were used as the input for a two-layer bidirectional long short-term memory (biLSTM).^18^ As was the case when fine-tuning SciBert, a CRF was used to ensure that the model only made valid predictions. While this approach has been shown to perform slightly worse in previous research,^6^ it was adopted here due to its relative similarity to other chemical NER systems that have performed well.^5,11^

**Figure 4:**
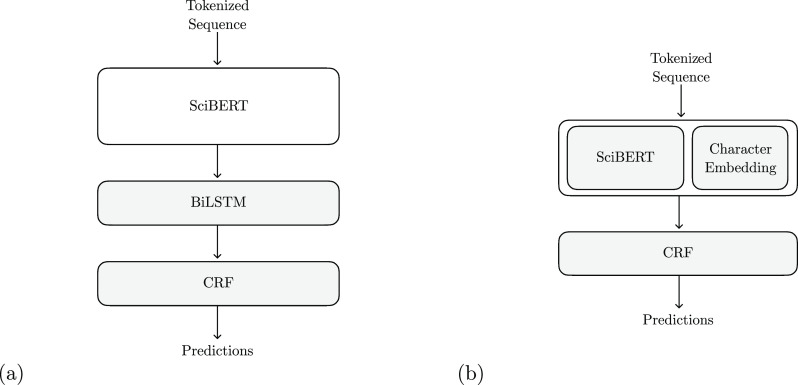
Structure of the NER model created by using the Bert vectors as contextualized embeddings (a) which are then fed into a bidirectional LSTM (BiLSTM). These contextual embeddings are supplemented by character embeddings in the variant (b). The shaded parts of the model were trained, while the unshaded parts were left frozen.

In addition to the NER model described above, a variant of this model was created where the SciBert vector was concatenated with character embeddings provided by a Convolutional Neural Network (CNN) before being fed into the biLSTM. While this approach of supplementing the output of a language model with character embeddings is used in other models such as ELMo,^19^ it is uncommon with Bert models. We nevertheless added this variant, as we hypothesized that the large number of unseen words seen in chemical NER could lead to the addition of character embeddings’ increasing performance. Both approaches are shown in Figure 4.

#### Evaluation

While the F1 score is normally seen as the most important metric for evaluating NER tasks, we focus more on the precision for the evaluation of our NER models since they are designed for IE. As the first step in an IE pipeline that generates databases, any decrease in precision for NER results in worse performance for every subsequent step of the pipeline. Above a certain level of recall, the gains for the IE pipeline, from being able to extract slightly more entities, are minimal, while the harm from extracting wrong entities is large. We define a reasonable level of recall as anything over 85%, and we manually picked the hyperparameters that afforded the highest precision on the development set given that they meet this level of recall.

### Data Sets

The models detailed in the Methodology section were trained on a corpus composed of samples from the CHEMDNER data set and the Matscholar data set, ensuring that the system was trained on a mix of sentences from both organic and inorganic chemistry.

#### CHEMDNER

The BioCreative IV CHEMDNER corpus consists of 84,355 chemical mention annotations across 10,000 abstracts, with an interannotator agreement of 91%.^4^ While documents were annotated and selected from across the entirety of chemical knowledge, the selection was such that the annotated chemical entities were biased toward organic ones. This can be seen in the disciplines from which papers were chosen for annotation: Biochemistry & Molecular Biology, Applied Chemistry, Medicinal Chemistry, Organic Chemistry, Physical Chemistry, Endocrinology & Metabolism, Chemical Engineering, Polymer Science, Pharmacology & Pharamacy, and Toxicology.

We compare our results on the CHEMDNER data set with those from the state-of-the-art *HanPaNe*+P model.^11^ This LSTM-based model utilizes word embeddings combined with character-level embeddings of the word and the sentence and achieves high performance via data augmentation and multitask learning.

#### Matscholar

In contrast to the CHEMDNER data set which strives to cover chemical mentions for a large part of chemistry, the Matscholar data set focuses on Materials Science and, as a result, contains mostly inorganic chemicals.^5^ Another key difference between the corpora is that the Matscholar data set contains a richer variety of named entities. While the CHEMDNER data set only contains annotations for chemicals, the Matscholar data set also contains other labels, such as material applications and material properties. The Matscholar data set consists of 800 annotated abstracts including 7,360 annotations of chemical names, making it a substantially smaller data set than the CHEMDNER data set. The creators of the data set report an interannotator agreement of 87.4%.

The state-of-the-art model for the Matscholar data set utilizes a Word2Vec^20^ embedding trained on scientific papers combined with character embeddings from a bidirectional LSTM to represent the sequence. This representation is fed into another bidirectional LSTM, with output constrained by a CRF.^5^

#### Data Set Variation

The differences between the two data sets can be seen not only in their aims but also quantitatively in the actual entities labeled. To investigate the differences, first, all of the chemical named entities from each entire data set were extracted. They were then normalized by lowercasing and removing any whitespace. As a result of this normalization, the CHEMDNER data set was found to have 17,488 unique entities, and the Matscholar data set was found to have 1,904 unique entities. Of these, 244 were found to be common to both data sets. This is a tiny fraction of the CHEMDNER data set and only 13% of the Matscholar data set, despite the comparison with a much larger data set.

This does not represent the varied nature of chemical named entities, in general; rather, it is an effect of the difference between the two data sets. For example, within the CHEMDNER data set where the training, development, and test sets are of roughly equal size, approximately a quarter of the named entities are shared between any two of these. Similar results can be obtained if we compare the development and test data sets of Matscholar, which are also the same size as each other. The fact that only 13% of the elements of the smaller data set were shared with elements of the larger data set captures the stark dissimilarity between the data sets, making the combined data set worthwhile.

#### Combined Data Set

To create an NER model that can work well for both inorganic and organic materials, these two disparate data sets were merged together to create a combined data set. In creating this combined data set, the Matscholar data set was stripped of all labels other than those for chemical named entities. The training and development data sets were redistributed within the CHEMDNER data set to follow a more standard 90:10 split before combining the data sets. Furthermore, the CHEMDNER data set was processed in the same manner as the Matscholar data set in that any tokens consisting of only numbers were replaced with the ⟨nUm⟩ token. Finally, the training data set was shuffled so that the model would encounter examples evenly from both data sets during training.

## Results and Discussion

### Tokenizer Choice

**Figure 5:**
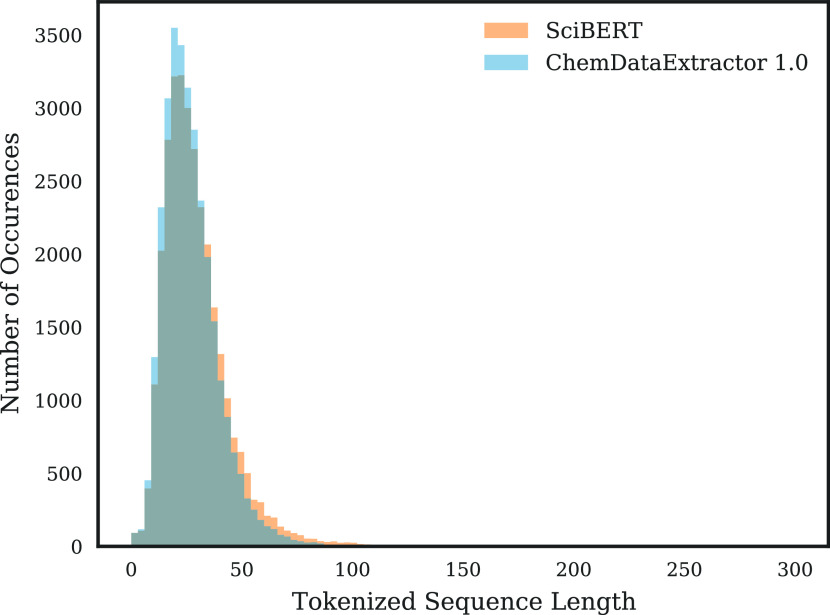
Distribution of tokenized sequence lengths for the CHEMDNER corpus.

The results of tokenizer testing can be seen in Table 1. Both of the tested tokenizers had their strengths, with the ChemDataExtractor 1.0 tokenizer yielding shorter tokenized sequences and the SciBert tokenizer yielding fewer partial chemical entities. However, the SciBert tokenizer was selected as it achieved a more than 5-fold reduction in partial chemical entities with a less than 2-fold increase in maximum tokenized sequence length. This reduction is significant as the 1,340 partial chemical entities represent almost 5% of the chemical entities labeled in the CHEMDNER data set, significantly increasing the potential performance of the NER system. Furthermore, despite the significant increase in the longest tokenized sequence length, the distribution of sequence lengths was actually relatively independent of the tokenizer choice, as illustrated in Figure 5.

**Table 1:** Results of Tokenization on the CHEMDNER Corpus

	no. of partial chemical entities	longest tokenized sequence length
ChemDataExtractor 1.0	1,340	**171**
SciBert	**218**	272

Qualitatively, both tokenizers failed at correctly tokenizing chemical entities such as “*Ser*845” and “rh*N-acetylgalactosamine 4-sulfatase*”, where the chemical entities “Ser” and “N-acetylgalactosamine 4-sulfatase” had no obvious boundaries with things that were not part of the chemical entity. In addition to these types of errors, the ChemDataExtractor 1.0 tokenizer would fail to tokenize things such as “S-transferase” and “GABA-benzodiazepine”, where the chemical entity was separated by a hyphen.

### NER Results

#### Using SciBert for Contextual Embeddings vs Fine-Tuning SciBert

A manual hyperparameter search was conducted for all NER models, which were trained using the Adam optimizer.^21^ The models with the best precision were kept provided they had achieved a recall greater than 85%. The performance of each model can be seen in Table 2.

**Table 2:** Performance Comparison on the Development Set of the Combined Data Set between the Different Model Variants

	precision	recall	F1
fine-tuned SciBert	**89.9**	86.9	88.3
ChemDataExtractor 1.0	86.6	82.8	84.7
SciBert embeddings only	86.9	90.3	88.5
SciBert + character embeddings	88.7	**90.7**	**89.66**

The fine-tuned SciBert model delivered the highest performance for our chosen metric, precision. The model that used SciBert vectors as contextual embeddings performed slightly worse in comparison, as shown in previous studies.^6,7^ While the fine-tuned model achieved a lower F1 score, this is due to our selection criteria which prioritizes precision. There were fine-tuned models with higher F1 scores than those achieved by the model using frozen SciBert vectors.

In contrast, the improvement in NER performance afforded by the addition of character embeddings is surprising. The performance is significantly better, with this NER model now surpassing the fine-tuned model in the F1 score. Unlike the difference between F1 scores described in the previous paragraph, this is not an artifact of selecting the models with the highest precision for each mode; even if the hyperparameters yielding the highest F1 score were to be picked for both types of models, the results would be the same, with the best performing fine-tuned SciBert model having an F1 score of 89.1.

#### Overall Results

Our final results on the test sets of the CHEMDNER and Matscholar corpora are presented in Table 3. Our model achieves the highest precision on the Matscholar data set and the second highest precision on the CHEMDNER data set. While our model did not surpass the state-of-the-art for the CHEMDNER data set, the key finding of this paper lies in that our model can perform to a high standard on *both* data sets, achieving a macro-averaged precision of 91.4%, 1.3% higher than the 90.1% macro-averaged precision of the state-of-the-art models for each data set.

**Table 3:** Results on the CHEMDNER Corpus and the Chemical NER Component of the Matscholar Corpus^a^

	CHEMDNER			Matscholar		
	precision	recall	F1	precision	recall	F1
our model (combined corpus)	92.0	87.5	89.7	**90.8**	85.4	88.0
our model (CHEMDNER only)	91.8	88.4	90.0	66.6	82.3	73.6
our model (Matscholar only)	24.2	24.0	24.1	87.6	91.8	89.6
ChemDataExtractor 1.0	89.1	86.6	87.8	63.3	58.4	60.7
*HanPaNe*+P^11^	**92.8**	**92.3**	**92.6**			
Matscholar^5^				87.3	**93.5**	**90.3**

a We could not access the *HanPaNe*+P^11^ and Matscholar^5^ systems, so their performance on out-of-domain fields is left blank.

Furthermore, by comparing the performance with the interannotator agreements of 91% and 87% for CHEMDNER and Matscholar, respectively, one can see that our model achieves close to human-level performance in chemical NER across both domains.

Qualitatively, many of the errors that are being seen from our system are of chemical entities that would be ambiguous to a human being as well. An example would be the phrase “single phase CuNd_2_O_4_-type tetragonal structures” from the Matscholar data set. Our system falsely labels CuNd_2_O_4_ as a chemical entity, while this is not labeled as a chemical entity in the data set due to its use in identifying a *type* of structure instead of being in reference to a chemical entity.

The system also qualitatively seems to have a relative weakness in recognizing longer, less formulaic names of chemical entities, especially those from the biological domain such as the false positives on “safranin” or “nucleotide” and the false positive on “parathormone” from the CHEMDNER data set; this was surprising given the amount of biological chemistry included in the CHEMDNER data set. This could be due to the relative variety of names in the biological domain.

What is surprising quantitatively is that training on both data sets results in only a small decrease or even an increase in the precision on the seen data set. This result was unexpected given that the models are now having to learn more, although it may be a product of the increased robustness afforded by training on such diverse data sets. However, while the precision of our NER model is higher when trained on both corpora, it seems to come at the cost of worse recall.

## Conclusions and Future Work

This paper presents a single NER system that performs competitively with the state-of-the-art across two different data sets that cover organic and inorganic chemical entities. We believe that this presents a substantial improvement for chemical NER systems, especially when used as a part of a real world IE pipeline, where documents may well involve chemical entities from many domains and having high performance across all of them is vital. The high score achieved by our NER system across two very different parts of chemistry also has positive implications for generalizability.

For future work, we believe that this approach could be extended to include more biomedical chemical entities, using data sets such as the CDR data set.^22^ This would also result in a much larger data set which could potentially even result in better performance for the existing data sets as well. Furthermore, a data augmentation approach may result in even higher performance, as demonstrated in prior research.^11^ Finally, some research into the unexpectedly high performance of SciBert vectors as contextual embeddings when combined with character embeddings may be warranted.

## Data and Software Availability

All of the scripts used to process the data that were employed to train the NER system are available online, as are the AllenNLP training configurations that were used to train the NER system.^23^ The final NER system is available independently as an AllenNLP model from our web site^24^ or as part of ChemDataExtractor 2.1, which is also available online.^25^ This includes the final tokenizer and model used (SciBert vocabulary with a fine-tuned SciBert model), but the other tokenizers and models mentioned in this paper are also available.^23^

## Supporting Information Available

Descriptions and statistics of other tokenizers trialed (PDF)
