# Supplementary material for: Single Model for Organic and Inorganic Chemical Named Entity Recognition in ChemDataExtractor
Source: J Chem Inf Model. 2022 Feb 24;62(5):1207–13. doi: 10.1021/acs.jcim.1c01199 (PMC9049593; doi:10.1021/acs.jcim.1c01199)
Supplement: Supplementary file 1 — ci1c01199_si_001.pdf [file ci1c01199_si_001.pdf]

A Single Model for Organic and Inorganic Chemical Named Entity Recognition in ChemDataExtractor - Supporting Information

Taketomo Isazawa† and Jacqueline M. Cole*,†,‡,¶

†*Cavendish Laboratory, Department of Physics, University of Cambridge, J. J. Thomson Avenue, Cambridge, CB3 0HE, UK*

‡*ISIS Neutron and Muon Source, STFC Rutherford Appleton Laboratory, Harwell Science and Innovation Campus, Didcot, Oxfordshire, OX11 0QX, UK*

¶*Department of Chemical Engineering and Biotechnology, University of Cambridge, West Cambridge Site, Philippa Fawcett Drive, Cambridge, CB3 0FS, UK*

E-mail: jmc61@cam.ac.uk

Other Tokenizers

The ChemDataExtractor 1.0 tokenizer and the SciBert SciVocab tokenizers were the main tokenizers tested in this work, but the same methodology was also used to test other tokenizers used for chemical NER. The results of this testing can be seen in Table S1. Each tokenizer not described in the paper is described in detail below.

WordTokenizer and FineWordTokenizer The WordTokenizer and FineWordTokenizer are other rule-based tokenizers that are included with ChemDataExtractor, with slightly different rules from the default ChemWordTokenizer. WordTokenizer omits any chemistry

Table S1: Results of tokenization on the CHEMDNER corpus.

	Number of partial chemical entities	Longest tokenized sequence length
ChemWordTokenizer[a]	1340	171
WordTokenizer	465	233
FineWordTokenizer	210	296
SpaCy	1067	169
Enhanced SpaCy	714	171
OSCAR (aggressive)	15	303
SciBert	218	272

[a]The ChemWordTokenizer is the default tokenizer used in ChemDataExtractor 1.0.

specific optimizations, aligning more with the Penn Treebank[1], while FineWordTokenizer tokenizes on any punctuation, leading to shorter tokens.

SpaCy The SpaCy[2] tokenizer is also a rule-based tokenizer with special handling of prefixes and suffixes. As can be seen in Table S1, it affords fewer partial chemical entity mentions than the ChemDataExtractor 1.0 tokenizer while also affording even shorter sequences.

Enhanced SpaCy The enhanced SpaCy tokenizer adds the ChemDataExtractor tokenizer rules on top of the SpaCy tokenized sequence to attempt to provide more chemistry-specific words that are tokenized well. This results in a further reduction in chemical entity mentions, accompanied by a small increase in tokenized sequence length.

OSCAR The OSCAR[3] tokenizer uses custom chemistry specific vocabulary to ensure that words such as "C-H" are not tokenized incorrectly. A Python reimplementation of this tokenizer[4] was used with the aggressive setting, which resulted in very long tokenized sequences (the longest sequences out of all tokenizers in the comparison), although it did also result in very few partial chemical entities.

References

(1) Marcus, M.; Santorini, B.; Marcinkiewicz, M. A. Building a large annotated corpus of English: The Penn Treebank. **1993**,

(2) Honnibal, M.; Montani, I. spaCy 2: Natural language understanding with Bloom embeddings, convolutional neural networks and incremental parsing. **2017**,

(3) Jessop, D. M.; Adams, S. E.; Willighagen, E. L.; Hawizy, L.; Murray-Rust, P. OSCAR4: a flexible architecture for chemical text-mining. *Journal of Cheminformatics* **2011**, *3*, 1–12.

(4) Corbett, P.; Boyle, J. Chemlistem: chemical named entity recognition using recurrent neural networks. *Journal of Cheminformatics* **2018**, *10*, 59.
